# Prevalence and related factors of TB/HIV co-infection among hospitalized children with tuberculosis in Southwest China

**DOI:** 10.3389/fcimb.2025.1571291

**Published:** 2025-06-11

**Authors:** Dong-Mei Wang, Qi An, Qing Yang, Yi Liao

**Affiliations:** ^1^ Department of Science and Education Division, Public Health Clinical Center of Chengdu, Chengdu, Sichuan, China; ^2^ Department of Clinical Laboratory Medicine, Chengdu Women’s and Children’s Central Hospital, School of Medicine, University of Electronic Science and Technology of China, Chengdu, China

**Keywords:** pediatric, tuberculosis, TB/HIV co infection, epidemiology, clinical characteristics

## Abstract

**Objectives:**

This study aimed to investigate the prevalence of TB/HIV co-infection in pediatric TB patients in southwest China and its associated variables.

**Methods:**

Pediatric TB patients were recruited from January 2014 to September 2024 in southwest China, based on etiology or clinical confirmation. Hospitalization records were extracted for each patient.

**Results:**

Among 2,607 pediatric TB patients with an average age of 9.58 ± 4.08 years, 39 (1.5%) were HIV-positive. The TB/HIV co-infection group male-to-female ratio was 2:1, higher than the TB-only group 1.19:1. The highest proportion of TB/HIV co-infection was in the 5-9 years age group (43.6%), while the 10-14 years age group accounted for the highest proportion of TB-alone cases (57.5%). In terms of population distribution, the Yi ethnic group had the highest proportion of TB/HIV co-infection cases (43.6%), while the Tibetan group had the highest proportion of TB-alone cases (51.1%). Extrapulmonary TB in the TB/HIV co-infection group primarily involved abdominal and pericardial sites, whereas the TB-alone infection group had more cases of lymphadenitis and pleural TB. The length of hospitalization (>14 days) in the TB/HIV co-infection group (74.4%) was significantly longer than in the TB-alone infection group (51.7%). Over the past 11 years, most pediatric TB/HIV co-infection cases were from the eastern-central and southern-central regions of Sichuan, particularly the southern Liangshan Yi Autonomous Prefecture. The number of children with TB-alone infections increased gradually during this period. No significant difference in the number of pediatric TB/HIV co-infection cases was observed over the 11 years.

**Conclusion:**

Pediatric TB/HIV co-infection in southwest China predominantly affects middle-aged and young boys, with a higher co-infection rate than the national average. The central and southern regions of Sichuan have a relatively high proportion of cases. Public health efforts should focus on strengthening awareness, screening, and early diagnosis of TB and HIV in children in high-risk areas to prevent further infections.

## Introduction

Human immunodeficiency virus (HIV) and tuberculosis (TB) are leading causes of infectious disease-related deaths worldwide. Limited reports exist on TB/HIV co-infection in children. According to the World Health Organization’s latest Global Tuberculosis Report 2024, approximately 1.3 million children and adolescents worldwide had TB in 2023, accounting for 12% of new global TB cases (WHO, 2024). The United Nations AIDS Programme estimates that 1.4 million children (aged 0-14 years) will be infected with HIV in 2024 (Joint United Nations Programme on HIV/AIDS, 2024). HIV is a major risk factor for TB, as it facilitates the progression of latent or recent *Mycobacterium tuberculosis* infections into active disease, thereby increasing the incidence of TB (Koch et al., 2017). The symptoms and clinical signs of these two diseases often overlap, complicating the diagnosis of TB in children. A meta-analysis of 29 TB/HIV co-infections in China indicates an average co-infection rate of 0.9% among Chinese adults with TB (Gao et al., 2010), with a lower rate of 0.22% in Fujian Province (Zhou et al., 2023). To address this issue, we conducted a study in Chengdu, the central city of southwest China, with a population of 21.4 million in 2023, of which 13.28% are children aged 14 and younger. This study, conducted at the Public Health Clinical Center (PHCC), a central unit for infectious disease management, aimed to estimate the prevalence of HIV/AIDS among hospitalized children with TB and compare factors associated with HIV-positive and HIV-negative pediatric TB patients, in order to provide data to support the clinical diagnosis and treatment of pediatric TB and TB/HIV co-infection patients.

## Methods

### Study design and population

**Figure 1:**
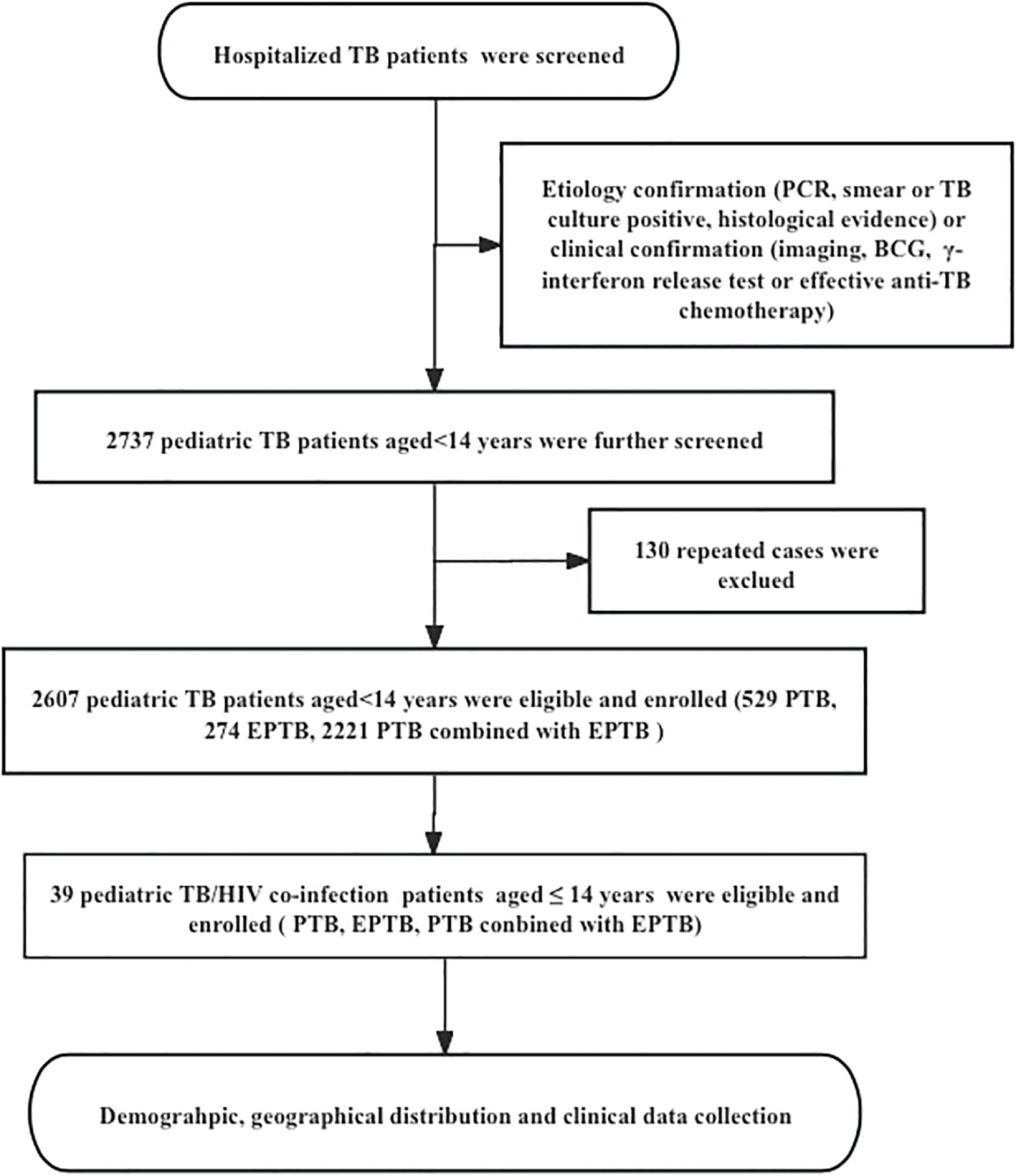
The flow diagram of our study. Demographic information and clinical data was reviewed from the Public Health Clinical Center of Chengdu, Sichuan, China; PTB, pulmonary tuberculosis; EPTB, extrapulmonary tuberculosis.

This is a observational descriptive study, pediatric TB patients were recruited from January 2014 to September 2024 at PHCC, based on etiology. Cases were defined as bacteriologically confirmed if a biological specimen was positive by smear microscopy, polymerase chain reaction (PCR), or culture, according to WHO guidelines. Clinical confirmation was used when etiology was not supportive, but diagnosis was based on imaging, Bacillus Calmette-Guérin (BCG) vaccination, γ-interferon release tests, or effective anti-TB chemotherapy in patients with clinical manifestations. Inclusion criteria (children aged ≤14 years with confirmed TB diagnosis) and exclusion criteria (repeated cases with multiple hospitalizations and cases with incomplete medical records). After excluding cases, a total of 2,607 pediatric TB patients aged ≤14 years were included in the study (**Figure 1**). Information from the hospital’s medical record management system and the information management system (HIS) was collected for each patient, including socio-demographic, clinical, therapeutic [e.g., use of antiretroviral therapy (ART) or TB treatment], geographic distribution of habitual residence, laboratory, and hospital outcome data etc.

### Diagnostic criteria

The diagnosis of pediatric TB was based on the Chinese Pulmonary Tuberculosis Diagnostic Criteria (WS 288–2017), the Chinese TB Clinical Diagnosis and Treatment Guidelines (Chinese Medical Association, 2005), and the updated WHO guidelines (World Health Organization (WHO), 2014). According to WHO guidelines, pulmonary tuberculosis (PTB) refers to TB disease involving the lung parenchyma and/or the tracheobronchial tree, including miliary TB, with or without a diagnosis of extrapulmonary tuberculosis (EPTB). All other sites of disease are classified as EPTB. Concurrent extrapulmonary and pulmonary TB (combined TB) was defined as any case of TB involving both the lungs and other organs. The diagnosis of TB was made through the evaluation of a specialist physician who evaluated the signs and symptoms and the results of laboratory tests, such as the presence of acid-fast bacilli in a sputum sample, X-ray compatible with active pulmonary TB, Positive molecular biological tests (such as PCR), positive sputum culture for *Mycobacterium tuberculosis*, etc. Acquired immunodeficiency syndrome (AIDS) was diagnosed based on the Chinese AIDS and HIV infection diagnostic criteria (WS293–2008) (From the Centers for Disease Control and Prevention1993 revised classification system for HIV infection and expanded surveillance case definition for AIDS among adolescents and adults, 1993). In the laboratory of PHCC, the HIV detection methods we carry out include screening experiments: colloidal gold immunochromatography, an Enzyme-Linked Immunisorbent Assay (ELISA), chemiluminescence etc. for detecting HIV-Ab. HIV infection was confirmed by Western blotting. Meanwhile, the viral content of HIV was detected by real-time fluorescence quantitative PCR.

### Ethical statement

This study was approved by the Ethics Committee of the Public Health Clinical Center (PHCC) of Chengdu (China) under the approval number YJ-K2023-08-01. As a retrospective study utilizing routinely collected patient data from the mandatory notification system, the Ethics Committee waived the requirement for informed consent.

### Statistical analysis

Data analysis was performed using STATA 17.0. Quantitative indicators are described using Mean ± SD, and according to the results of normality analysis, analysis of variance is used for inter group comparisons that conform to normal distribution, while non parametric tests are used for comparisons that do not conform to normal distribution. Classification indicators are described using frequency (percentage), and inter group comparisons are conducted using chi square test/Fisher test, and a *P* value < 0.05 was considered statistically significant.

## Results

### Demographic and clinical characteristics

The pediatric TB patients in this study, 54.3% (1,416/2,607) were male and 45.7% (1,191/2,607) were female, resulting in a male-to-female ratio of 1.19:1. Among the children with TB in this study, the TB/HIV co-infection rate was 1.5% (39/2,607). In the TB/HIV co-infection group, males (66.7%, 26/39) outnumbered females (33.3%, 13/39), and the male-to-female ratio of 2:1 was higher than that of the TB-only group (1.19:1). The mean age of the 2,607 pediatric TB patients was 9.58 ± 4.08 years (range: 18 days to 14 years). Among the patients, 19.8% were aged 0–4 years, 23.1% were aged 5–9 years, and 57.2% were aged 10–14 years. In the TB/HIV co-infection group, the highest proportion was in the 5–9 years age group (43.6%, 17/39), whereas the TB-only group had the highest proportion in the 10–14 years age group (57.5%, 1477/2568) (χ² =11.292, *P* =0.004) (**Table 1**). Regarding population distribution, the Yi ethnic group had the highest proportion of TB/HIV co-infection cases in children (43.6%, 17/39), while, the Tibetan group had the largest proportion of TB-only cases (51.1%, 1311/2568), with a statistically significant difference (χ²= 60.622, *P* < 0.001). The average length of hospital stay for children in the TB/HIV co-infection group was significantly longer than that for the TB-only infection group (51.7%, 1327/2568), with a statistically significant difference (χ² = 8.425, *P* = 0.012) (**Table 1**). Among the pediatric TB/HIV co-infection patients, 20.5% had a decreased CD4 T-cell count. Laboratory results indicated that 23 patients (59.0%) had an elevated erythrocyte sedimentation rate (ESR), 21 patients (53.8%) had varying degrees of anemia, 10 patients (25.6%) had malnutrition, and 26 patients (66.7%) had leukopenia during treatment (**Table 2**).

**Table 1:** Demographic, clinical characteristics of pediatric TB and TB/HIV co-infection cases in southwest of China.

Variable	TB/HIV co-infection (n=39) %		TB only (n=2568) %		All (n=2607) %		χ ^2^	*P*
Sex								
Male	26	66.7	1390	54.1	1416	54.3	2.434	0.119
Female	13	33.3	1178	45.9	1191	45.7		
Age group (years)								
Mean ± SD; years	7.33 ± 3.39		9.56 ± 4.03		9.58 ± 4.08			
0–4	9	23.1	507	20	516	19.8	11.292	0.004
5-9	17	43.6	584	22.7	601	23.1		
10-14	13	33.3	1477	57.5	1490	57.2		
Race/ethnicity		0.0				0.0		
Han nationality	15	38.5	1007	39.2	1022	39.2	60.622	<0.001
Tibetan	7	17.9	1311	51.1	1318	50.6		
Yi	17	43.6	220	8.6	237	9.1		
Others			30	1.2	30	1.2		
One or both parents to AIDS	20	51.3	/	/	/	/		
TB site								
Pulmonary	10	25.6	466	18.1	476	18.3	6.830	0.031
Extra-pulmonary	9	23.1	311	12.1	320	12.3		
Both	20	51.3	1791	69.7	1811	69.5		
Complication								
Pulmonary PCP infection	1	2.6		/	1	0.0	/	/
Epilepsy	1	2.6	100	3.9	101	3.9		
Hyperuricemia	7	17.9	342	13.3	349	13.4		
Rash	1	2.6	15	0.6	16	0.6		
Hepatic Insufficiency	11	28.2	349	13.6	360	13.8		
HBV	3	7.7	21	0.8	24	0.9		
Length of stay								
<7	4	10.3	328	12.8	332	12.7	8.425	0.012
7-14	6	15.4	913	35.6	919	35.3		
>14	29	74.4	1327	51.7	1356	52.0		
Disease outcome*								
Get better	37	94.9	2483	96.7	2520	96.7	/	/
Cure	0	0.0	31	1.2	31	1.2		
Unhealed	1	2.6	26	1.0	27	1.0		
Death	0	0.0	13	0.5	13	0.5		
Others	1	2.6	15	0.6	16	0.6		

*Get better, discharged after their condition improved and was controlled; Cure, treatment completed; Unhealed, after treatment, the patient's disease symptoms did not show significant improvement, and there might even be a situation where worsened; Death, the patient died during hospitalization; Others, transfer out or discharged without medical advice, etc.

**Table 2:** Laboratory findings of pediatric TB/HIV co-infection cases in southwest of China (n = 39).

Variable	Frequency	Percent (% )
Blood results		
Anemia ^a^	21	53.8
mild anemia	10	25.6
moderate anaemia	10	25.6
severe anemia	1	2.6
ESR (Female > 20, male > 15 mm/hour)	23	59.0
HIV RNA (copies/mL), median (IQR)	3.27E+5 (1.26E+5 - 6.04E+5)	
White blood cell counts <4.00*10^9^/L	26	66.7
Platelet (<100 ×10^9/L)	5	12.8
CD4 count(<400 cells/mL)	8	20.5
C-Reactive protein (> 6 mg / L)	18	46.2
Malnutrition	10	25.6

ESR erythrocyte sedimentation rate; ^a^0.5–4.99 yrs. Hemoglobin < 110 g /L, 5–11.99 yrs. Hemoglobin < 115 g/L, 12–14.99 yrs. Hemoglobin < 120 g/L (McLean et al., 2009).

### Type distribution and age proportion among PTB, EPTB, and combined TB groups

**Figure 2:**
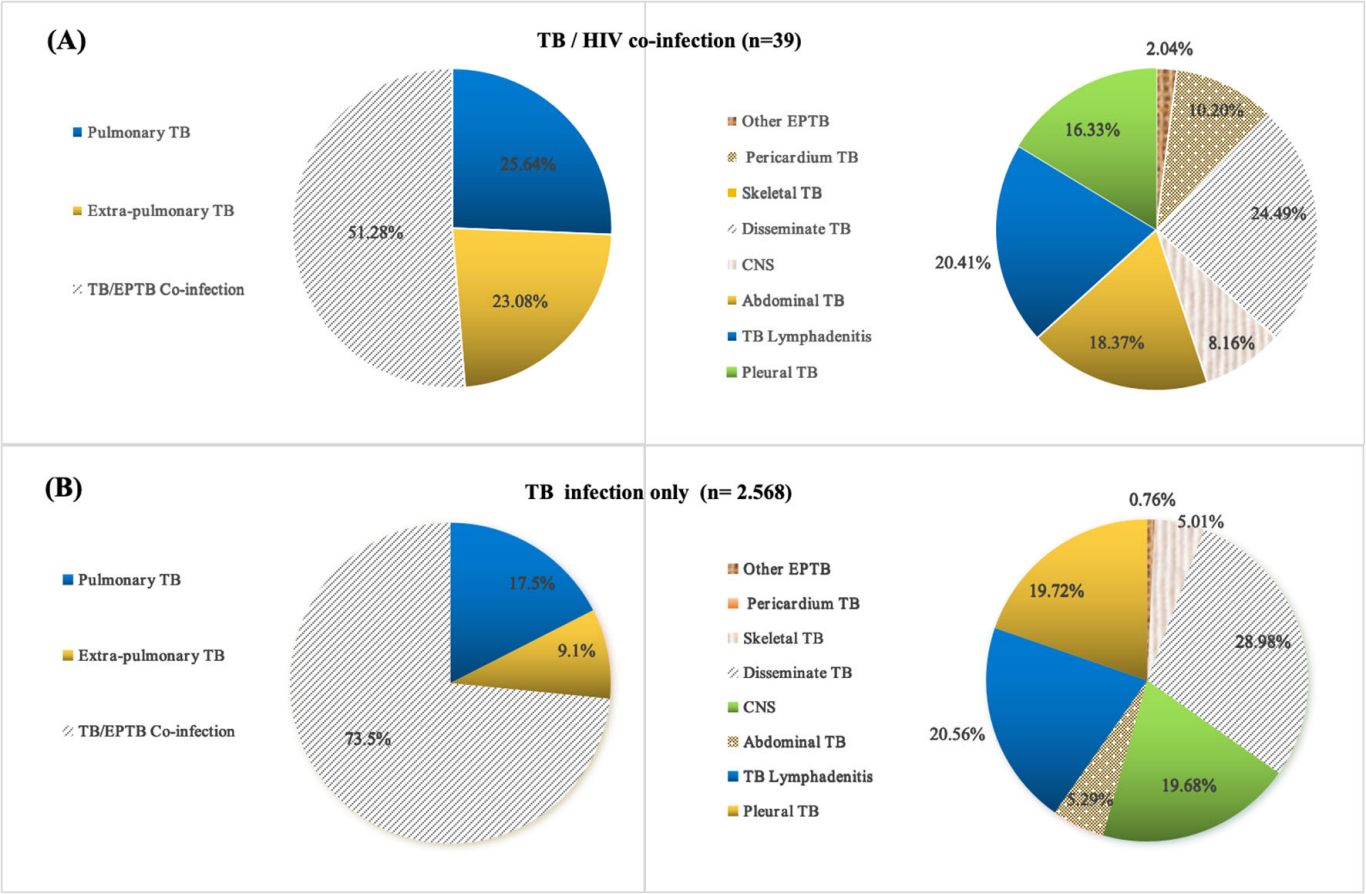
Location and distribution of TB lesions in pediatric TB and TB/HIV co-infection cases; **(A)** Tissue type distribution of pediatric TB/HIV co-infection cases; **(B)** Tissue type distribution of pediatric TB infection only cases; TB, tuberculosis; PTB, pulmonary tuberculosis; EPTB, extrapulmonary tuberculosis.

Among the pediatric TB/HIV co-infection patients, 25.64% (10/39) had pulmonary TB (PTB), 23.08% (9/39) had extrapulmonary TB (EPTB), and 51.28% (20/39) had combined TB. The most common form of pediatric TB/HIV co-infection was disseminated TB (24.49%, 10/39), followed by lymphatic TB (20.41%, 8/39), abdominal TB (18.37%, 7/39), pleural TB (16.33%, 6/39), pericardial TB (10.20%, 4/39), central nervous system (CNS) TB (8.16%, 3/39), and other forms of EPTB (2.04%, 1/39) (**Table 1**) (**Figure 2A**). In the TB-only group, 17.5% (448/2568) had PTB, 9.1% (233/2568) had EPTB, and 73.5% (1887/2568) had combined TB. The most common form of pediatric TB was disseminated TB (28.98%, 744/2568), followed by lymphatic TB (20.56%, 528/2568), pleural TB (19.72%, 506/2568), CNS TB (19.68%, 505/2568), abdominal TB (5.29%, 136/2568), skeletal TB (5.01%, 129/2568), and other forms of EPTB (0.76%, 20/2568) (**Figure 2B**).

### Disease incidence trend and geographical distribution

**Figure 3:**
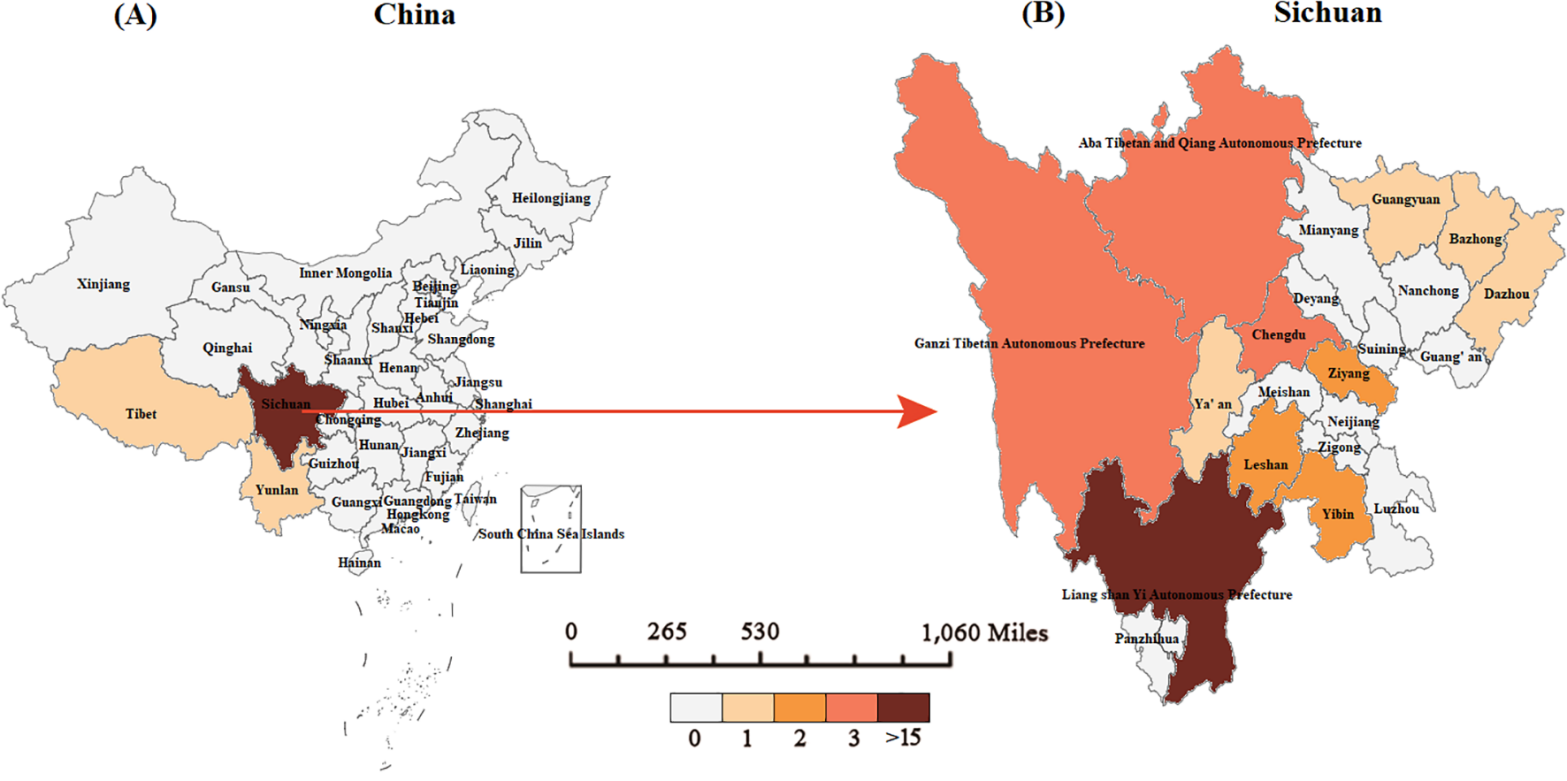
Geographical distribution of pediatric TB/HIV co-infection cases in our study. **(A)** The geographical distribution of pediatric TB/HIV cases in China. **(B)** The geographical distribution of pediatric TB/HIV co-infection cases in Sichuan.

**Figure 4:**
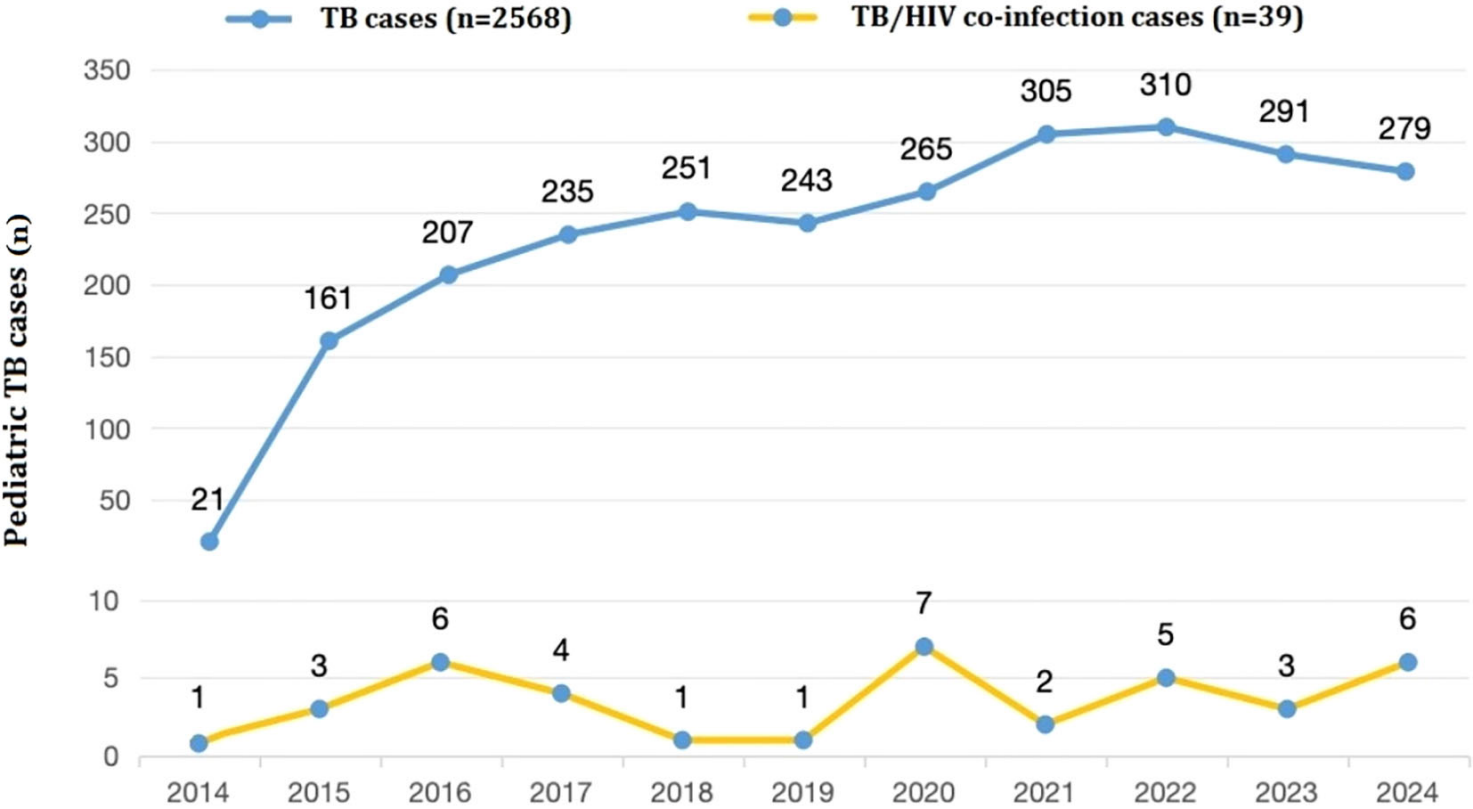
Numbers of pediatric TB or TB/HIV co-infection patients presenting each year between 2014 and 2024 (n =2,607).

The 39 pediatric TB/HIV co-infection patients diagnosed and treated at PHCC between January 2014 and September 2024 were matched to a 1:100,000 digital map of China using Python 3.7. The geographical distribution map indicated that over the 11-year period, two of the pediatric TB/HIV co-infection patients were from neighboring cities outside Sichuan Province (Tibet and Guizhou), while the remaining cases were from within Sichuan Province, primarily from the central and southern regions, with the highest number of cases in the Liangshan Yi Autonomous Prefecture (**Figure 3**). No significant difference was observed in the incidence rate of pediatric TB/HIV co-infection cases over the same period. The incidence rate of children infected with TB alone gradually increased over the past 11 years (χ² = 0.356, *P* = 0.012) (**Figure 4**).

### Treatment and outcomes

The basic anti-TB treatment plan for the 2,568 patients with pediatric TB was 2HREZ/6HR. For TB meningitis or disseminated TB, the treatment course is usually extended to 9 to 12 months. However, when evaluation of the efficacy of the anti-TB treatment plan indicated poor efficacy or a definite diagnosis of drug resistance was made, the treatment regimen could be adjusted. Among the 39 children with TB/HIV co-infection, basic anti-TB treatment plan, a 2-month intensive HR (rifampicin/rifabutin) EZ and a 4-6 month consolidation HR (rifampicin/rifabutin) are still adopted. Among the 39 cases, 21 cases had received or were receiving ART. The treatment plan with the highest frequency of use was abacavir (ABC)+lamivudine(3TC)+ritonavir-boosted lopinavir (LPV/r) (10 cases), followed by zidovudine (AZT)+3TC+efavirenz (EFV) (4 cases), AZT+EFV (3 cases), ABC+3TC+EFV (2 cases) and ABC+3TC+dolutegravir (DTG) (2 cases). Some cases may suspend medication or adjust the treatment plan during the medication period due to allergy or drug resistance. Among the 39 children with TB/HIV co-infection, 37 were discharged after their condition improved and was controlled, while 2 patients requested early discharge for personal reasons.

## Discussion

TB in children is a crucial indicator for evaluating the effectiveness of national TB prevention and control measures. In recent years, although public awareness of pediatric TB has increased, the subtlety of its signs and symptoms has posed significant challenges to clinical diagnosis and treatment. HIV infection is a major risk factor for TB (Koch et al., 2017), as it leads to immune suppression, increasing both the incidence and complexity of TB. The co-infection of these two diseases in children further complicates diagnosis and treatment. Limited data are available regarding the HIV infection rate among pediatric TB patients, and existing information is difficult to interpret due to diagnostic challenges, underreporting, and population selection bias. Therefore, understanding the burden of TB/HIV co-infection in children in developing countries is critical. It can help address gaps in transmission and prevention mechanisms for these chronic diseases and enable more efficient allocation of limited resources for prevention, diagnosis, and treatment. With this in mind, this study included all pediatric TB cases with clinical diagnoses (age ≤ 14) hospitalized at PHCC, the centralized infectious disease management unit in Chengdu, a central city in southwest China, between January 2014 and December 2024. The aim was to estimate the prevalence of HIV among hospitalized children with TB and compare patient-related factors between HIV-positive and HIV-negative pediatric TB cases.

Among the children with TB in this study, the TB/HIV co-infection rate was 1.5%. As there are few reports on pediatric TB/HIV co-infection, comparisons with international data are useful. Among Japanese adults, the TB/HIV co-infection rate is reported to be 0.2–0.3% (Kawatsu et al., 2022), while in Brazilian children it is higher at 7% (Miranda et al., 2011). In contrast, a higher co-infection rate of 55.9% was reported in a study of PTB-confirmed cases in South Africa (Naidoo et al., 2013). The variation in TB/HIV co-infection prevalence across studies may be attributed to differences in TB prevalence, socio-economic status, geographical factors, and laboratory testing methods. Higher rates of TB and HIV in certain regions may contribute to more frequent co-infections. The co-infection rate among children in this study is slightly higher than the national average for adults, which could be linked to the elevated TB (Zuo et al., 2020) and HIV infection rates in southwest China (He, 2021; Shama et al., 2022). Therefore, special attention should be given to the central and southern regions of Sichuan, particularly the Liangshan Yi Autonomous Prefecture, which has the largest number of cases. This highlights the need for enhanced HIV screening and increased TB screening in areas with high HIV prevalence, which would significantly impact the prevention, control, diagnosis, and treatment of both diseases.

In this study, the proportion of TB/HIV co-infection was higher in boys than in girls, with the majority of cases occurring in middle-aged and older children. In contrast, the group infected with TB alone predominantly consisted of older children. The male-to-female ratio in this study aligns with findings from a study on TB/HIV co-infection in children in Brazil (Miranda et al., 2011). Over half of the children with TB/HIV co-infection had parents infected with AIDS, and nearly half of the cases were from the high-incidence area of AIDS in Liangshan Yi Autonomous Prefecture, southern Sichuan. This suggests that vertical transmission and close contact between mother and child may serve as risk factors for exposure to TB/HIV co-infection. Regarding ethnic distribution, the Yi ethnic group had the highest proportion of TB/HIV co-infection cases among children, while the Tibetan ethnic group had the highest proportion of TB-only cases. In previous studies on tuberculosis in southwest China, we identified ethnic minority areas in western Sichuan, particularly Tibetan regions, as high-incidence areas for TB (Wang et al., 2020; Wang et al., 2023; Wang et al., 2024). This study further emphasizes the unique geographical distribution of TB/HIV co-infection cases in the central and southern parts of Sichuan, with Liangshan Yi Autonomous Prefecture in the south being a focal point. The government should intensify prevention and control efforts in these key areas.

Malnourished children represent a high-risk group for both TB and AIDS. Severe malnutrition is strongly correlated with a higher mortality rate in children infected with TB and HIV (Kebede, 2022; Vonasek et al., 2022; Ockenga et al., 2023). The risk of complications is further exacerbated when TB/HIV co-infection occurs, making the interplay between these diseases a critical factor in improving children’s health outcomes. In this study, more than half of the children with TB/HIV co-infection exhibited varying degrees of anemia and low peripheral blood white blood cell counts. Additionally, their hospitalization duration was significantly longer compared to those with TB alone. Research indicates that nutritional intervention can significantly improve the cure rate for tuberculosis (Sinha et al., 2021) and greatly enhance the quality of life and survival rate for individuals with HIV infection (Duggal et al., 2012). Therefore, alongside targeted screening for TB and AIDS cases, it is essential to strengthen nutritional support and treatment for malnourished children, particularly in high-risk areas, to improve their nutritional status.

There is an international agreement to start ART in all children infected with HIV independently of clinical signs or CD4 cell count or percentage (Beghin et al., 2018). The treatment plan for children with TB/HIV co-infection needs to comprehensively consider the synergistic effect of anti-tuberculosis and ART, drug interactions, and the immune status of the children.

The guidelines of WHO (UNAIDS,) and US NIH (Canals et al., 2018) recommend the use of the ABC+3TC+LPV/r regimen for AIDS cases ≤3 years. PENTA guidelines hold that this protocol is also applicable to children aged 3-6 years (WHO Library Cataloguing-in-Publication Data, 2016). Among the cases of children with TB/HIV co-infection in our study, nearly half of the cases receiving ART used the ABC+3TC+LPV/r regimen.

In children younger than 3 years, LPV/r-based regimen is preferred to nevirapine based regimen from 2 weeks of life. It confers reduced risk of discontinuing treatment, virological failure due to non-nucleoside reverse-transcriptase inhibitors resistance (Kuhn et al., 2014). LPV/r has been studied in both antiretroviral-naive and antiretroviral-experienced children and has demonstrated durable virologic activity and low toxicity (Sáez-Llorens et al., 2003; Chadwick et al., 2011). Therefore, it is recommended that the ABC+3TC+LPV/r regimen be the first choice for young children. For older children(≥3 years to <12 years of age), WHO (UNAIDS,) and PENTA guidelines (WHO Library Cataloguing-in-Publication Data, 2016) recommend the use of regimens such as ABC/AZT+3TC+EFV, ABC +3TC+EFV or ATV/r.

Our study has several limitations. First, the cases in this study were limited to hospitalized children with TB at PHCC, a centralized infectious disease management unit in Chengdu, southwestern China. TB/HIV co-infection was not investigated in children with HIV infection concurrently. Consequently, the reported HIV positivity rate among children with TB in this study may underestimate the actual prevalence of TB/HIV co-infection in the broader pediatric population of southwest China. Furthermore, because routine HIV screening is not fully implemented in children during TB treatment, particularly in outpatient settings, it is challenging to determine whether the observed increase in HIV positivity among hospitalized cases reflects a true rise in TB/HIV co-infection or if HIV testing is more concentrated in high-risk groups.

## Conclusions

The TB/HIV co-infection population among hospitalized children in Southwest China is predominantly composed of middle-aged and young boys, with a co-infection rate higher than the domestic average. A significant proportion of cases are concentrated in the central and eastern parts of Sichuan, as well as the central and southern regions. Childhood TB/HIV co-infection remains a critical public health issue in southwestern China. Therefore, efforts to prevent and treat both HIV infection and TB should place greater emphasis on TB/HIV co-infection. Public health awareness and education should be strengthened to improve self-protection and health levels, with a focus on preventing both HIV infection and TB.

## Data Availability

The original contributions presented in the study are included in the article/supplementary material. Further inquiries can be directed to the corresponding author/s.
